# Supplementary material for: ADHD symptoms are associated with the modular structure of intrinsic brain networks in a representative sample of healthy adults
Source: Netw Neurosci. 2019 Apr 1;3(2):567–88. doi: 10.1162/netn_a_00083 (PMC6497005; doi:10.1162/netn_a_00083)
Supplement: Supplementary file 1 [file netn-03-567-s001.pdf]

Hilger, K. & C. J. Fiebach. (2019). Supporting information for "ADHD Symptoms are Associated with the Modular Structure of IntrinsicvBrain Networks in a Representative Sample of Healthy Adults. *Network Neuroscience*, *3*(2), 567–588. https://doi.org/10.1162/netn_a_00083

ADHD-Symptoms in Healthy Adults are Associated with the Modular Structure of Intrinsic Brain Networks

Kirsten Hilger[1,2*], Christian J. Fiebach[1,2,3]

[1] Department of Psychology, Goethe University Frankfurt, Frankfurt am Main, Germany
[2] IDeA Center for Individual Development and Adaptive Education, Frankfurt am Main, Germany
[3] Brain Imaging Center, Goethe University Frankfurt, Frankfurt am Main, Germany

Network Neuroscience

SUPPLEMENTARY MATERIAL

* Corresponding author:

Dr. Kirsten Hilger
Goethe University
Department of Psychology
Theodor-W.-Adorno-Platz 6, PEG
D-60323 Frankfurt am Main
Phone: +49 (0)69 / 798 - 35345
hilger@psych.uni-frankfurt.de

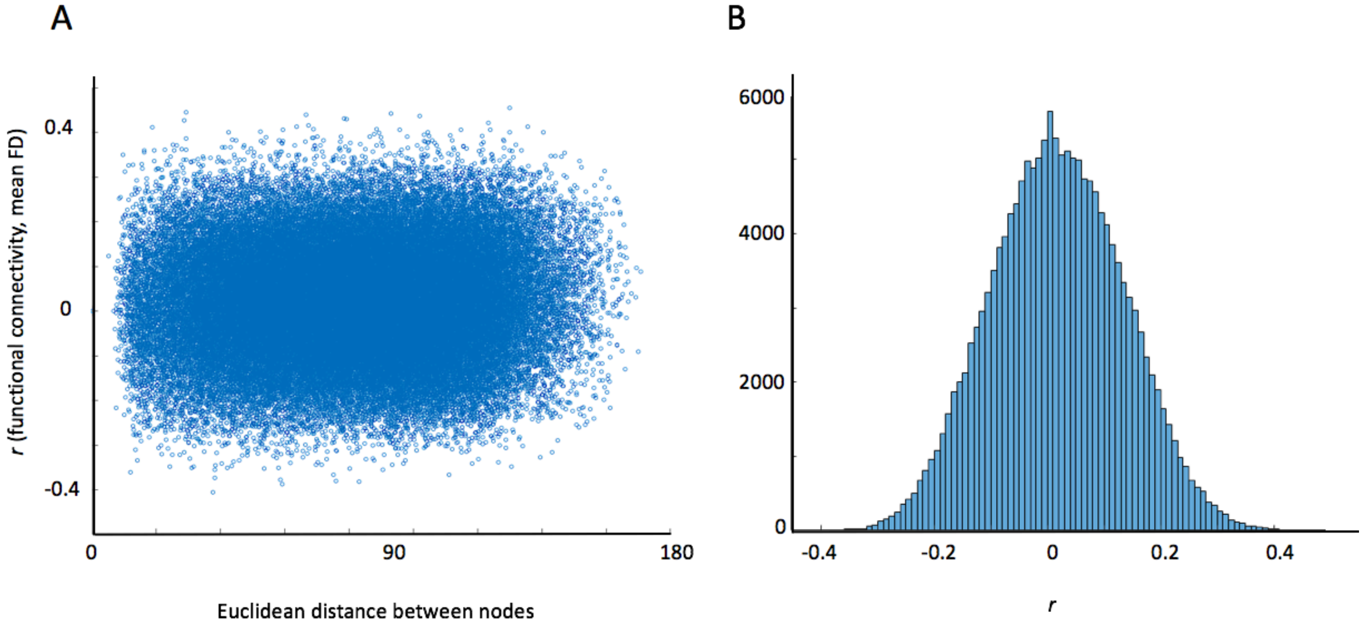

Supplementary Figure S1. Check for distance-dependent influences of in-scanner head motion on functional connectivity values. A, Scatterplot illustrating the correlation between each edge's functional connectivity strength and mean framewise displacement (*y*-axis) in dependency of Euclidean distance between the respective nodes of this edge (in mm, *x*-axis). B, Histogram of the correlation scores for the association between mean framewise displacement and functional connectivity values. FD, mean framewise displacement; *r*, Pearson correlation. Functional connectivity values are based on the 400-node parcellation of Schaefer et al. (2018).

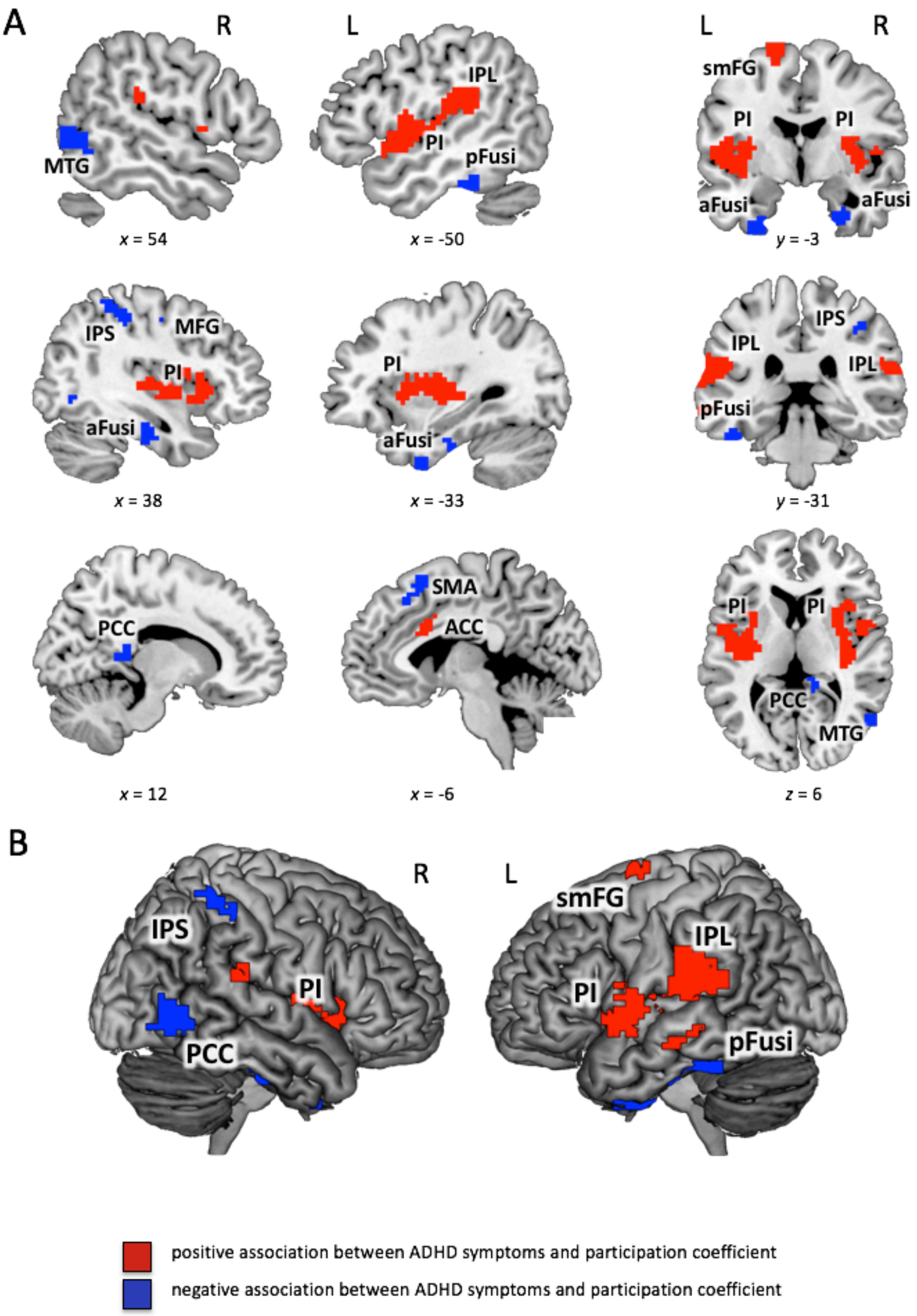

Supplementary Figure S2. Significant associations between Conners' ADHD Index and participation coefficient controlled for number of low-motion frames (rather than mean framewise displacement; see Post-Hoc Analyses in the Results Section of the Main Text, and Supplementary Table S6). *Participation coefficient* p_i (see Methods for details) was calculated for binarized and proportionally thresholded graphs using five thresholds (graphs were defined by the top 10%, 15%, 20%, 25%, or 30% of strongest edges). Input for analyses were the individual mean maps for *participation coefficient* p_i, which were calculated by averaging across these five thresholds for each participant separately. Statistic parametric maps of *participation coefficient* p_i are shown at a voxel-level threshold of $p < .005$ (uncorrected) combined with a cluster-level threshold of $k > 26$ voxels, corresponding to an overall family-wise error corrected threshold of $p < .05$ (see Methods). **(A)** Slice view; the *x*-, *y*-, and *z*-coordinates represent coordinates of the Montreal Neurological Institute template brain (MNI152).

(B) Render view; projection to the surface of the brain, search depth 12 voxels. PI, posterior insula; IPL, inferior parietal lobe; IPS, intraparietal sulcus; ACC, anterior cingulate cortex; MFG, middle frontal gyrus; SMA, supplementary motor area; aFusi, anterior fusiform gyrus; pFusi, posterior fusiform gyrus; PCC, posterior cingulate cortex; MTG, middle temporal gyrus; smFG, superior medial frontal gyrus.

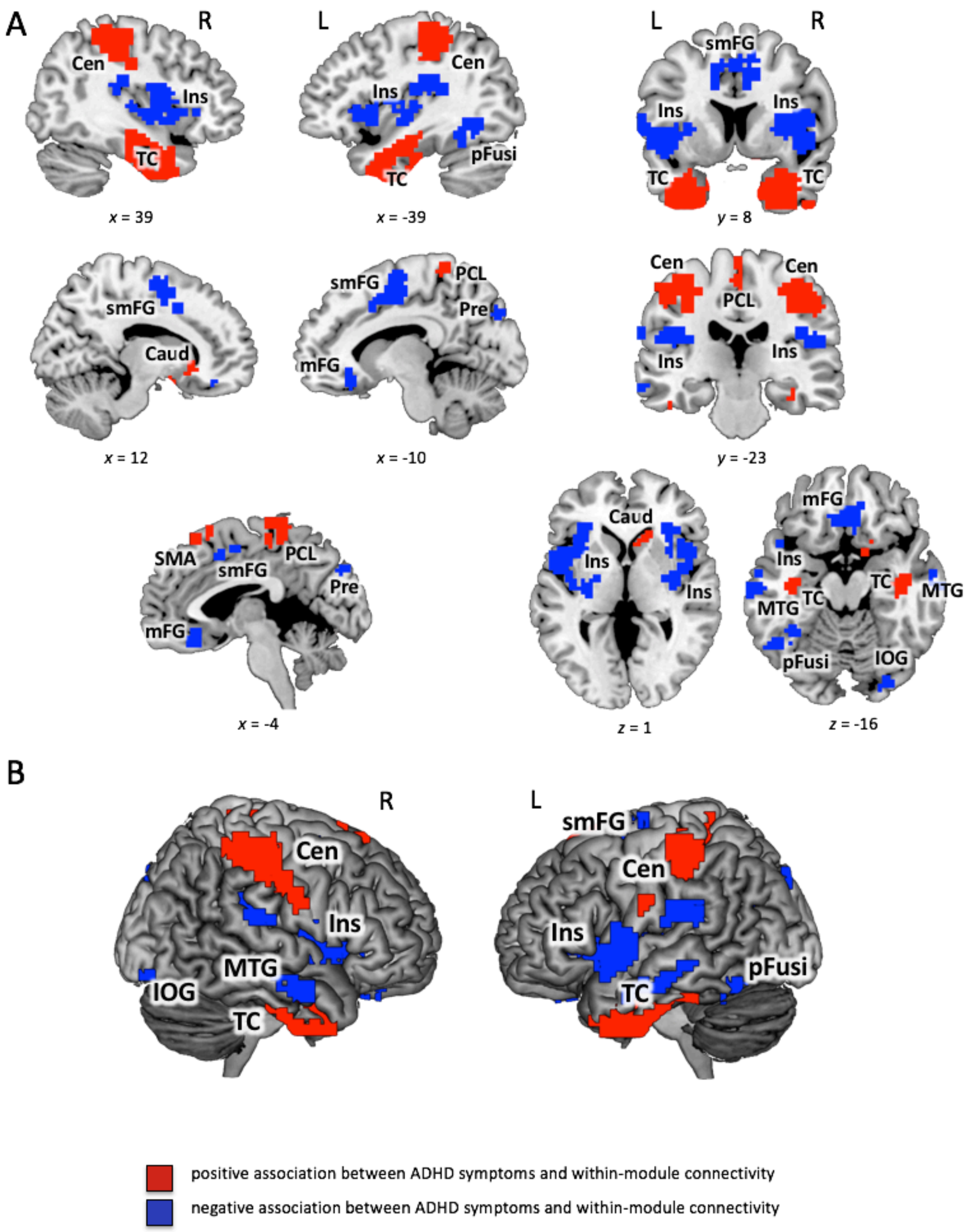

Supplementary Figure S3. Significant associations between Conners' ADHD Index and within-module degree controlled for number of low-motion frames (rather than mean framewise displacement; see Post-Hoc Analyses in the Results Section of the Main Text, and Supplementary Table S7). *Within-module degree* z_i (see Methods for details) was calculated for binarized and proportionally thresholded graphs using five thresholds (graphs were defined by the top 10%, 15%, 20%, 25%, or 30% of strongest edges). Input for analyses was the individual mean maps for *within-module degree* z_i, which were calculated by averaging across these five thresholds for each participant separately. Statistic parametric maps of *within-module degree* z_i are shown at a voxel-level threshold of $p < .005$ (uncorrected) combined with a cluster-level threshold of $k > 26$ voxels, corresponding to an overall family-wise error corrected threshold of $p < .05$ (see Methods). **(A)** Slice view; the *x*-, *y*-, and *z*-coordinates represent coordinates of the Montreal Neurological Institute template brain (MNI152). **(B)** Render view; projection to the surface of the brain, search depth 12 voxel. TC, temporal cluster comprising also amygdala,

hippocampus, and parts of fusiform gyrus; Cen, central cluster spreading across central and postcentral sulci from precentral gyri and postcentral gyri to the inferior parietal lobes (comprising supramarginal gyri and anterior parts of intraparietal sulci); PCL, paracentral lobule; mFG, medial frontal gyrus; Ins, insular cluster comprising also parts of putamen, superior temporal gyrus, inferior frontal gyrus and inferior parietal lobe; MTG, middle temporal gyrus; pFusi, posterior fusiform gyrus; Pre, precuneus; IOG, inferior occipital gyrus; smFG, superior medial frontal gyrus; SMA, supplementary motor area; Caud, caudate nucleus.

Supplementary Table S1. Correlations between CAARS subscales and whole-brain characteristics of modular network organization

	$r_{part.}$	$p_{part.}$
Inattention/Memory Problems		
global modularity	.051	.387
number of modules	-.050	.400
average module size	.043	.471
variability in module size	-.033	.579
Hyperactivity/Restlessness		
global modularity	.031	.596
number of modules	.007	.912
average module size	-.024	.681
variability in module size	-.010	.863
Impulsivity/Emotional Lability		
global modularity	.082	.166
number of modules	.037	.537
average module size	-.055	.356
variability in module size	-.019	.747
Self-Concept Problems		
global modularity	.147	.013
number of modules	-.062	.300
average module size	.061	.307
variability in module size	.118	.046

$r_{part.}$, Pearson's correlation coefficient for the partial correlation controlling for effects of age, sex, handedness, mean framewise displacement, and Full Scale Intelligence Quotient; $p_{part.}$, p-value of significance for the partial-correlation.

Supplementary Table S2. Correlations between CAARS subscales and proportions of node type within the whole brain

	$r_{part.}$	$p_{part.}$
Inattention/Memory Problems		
ultra-peripheral nodes	.053	.375
peripheral nodes	.024	.686
non-hub connector nodes	-.019	.751
non-hub kinless nodes	-.07	.239
provincial hubs	.025	.675
connector hubs	.009	.886
kinless hubs	-.096	.107
Hyperactivity/Restlessness		
ultra-peripheral nodes	.061	.307
peripheral nodes	-.026	.658
non-hub connector nodes	.010	.871
non-hub kinless nodes	-.006	.916
provincial hubs	-.042	.480
connector hubs	.048	.418
kinless hubs	-.074	.213
Impulsivity/Emotional Lability		
ultra-peripheral nodes	.115	.052
peripheral nodes	-.038	.518
non-hub connector nodes	.025	.675
non-hub kinless nodes	-.038	.52
provincial hubs	-.015	.800
connector hubs	.003	.956
kinless hubs	-.063	.285
Self-Concept Problems		
ultra-peripheral nodes	.042	.480
peripheral nodes	.068	.251
non-hub connector nodes	-.127	.032
non-hub kinless nodes	-.080	.180
provincial hubs	.015	.802
connector hubs	-.026	.656
kinless hubs	.034	.570

$r_{part.}$, Pearson's correlation coefficient for the partial correlation controlling for effects of age, sex, handedness, mean framewise displacement, and Full Scale Intelligence Quotient; $p_{part.}$, *p*-value of significance for the partial-correlation.

Supplementary Table S3. Correlations between Conner's ADHD Index and functional connectivity strength within/between canonical brain networks

	VIS	SOM	DAN	VAN	LIM	FPN	DMN
**VIS**	.011<br>(.847)	-	-	-	-	-	-
**SOM**	.012<br>(.833)	.007<br>(.901)	-	-	-	-	-
**DAN**	.016<br>(.782)	.016<br>(.784)	.021<br>(.717)	-	-	-	-
**VAN**	.035<br>(.552)	.024<br>(.684)	.107<br>(.069)	.031<br>(.591)	-	-	-
**LIM**	.024<br>(.675)	.004<br>(.939)	.043<br>(.463)	.084<br>(.635)	-.010<br>(.866)	-	-
**FPN**	-.015<br>(.794)	-.011<br>(.848)	.028<br>(.635)	.050<br>(.395)	-.022<br>(-.702)	.034<br>(.561)	-
**DMN**	-.086<br>(.142)	-.092<br>(.118)	-.102<br>(.084)	-.061<br>(.304)	-.044<br>(.449)	-.017<br>(.775)	.113<br>(.056)

Pearson's correlation coefficient for the partial correlation between functional connectivity strength and ADHD symptoms controlling for effects of age, sex, handedness, mean framewise displacement, and Full Scale Intelligence Quotient; corresponding *p*-values are depicted in brackets; VIS, visual network; SOM, somato-motor network, DAN, dorsal attention network, VAN, ventral attention network, LIM, limbic network, FPN, frontoparietal network, DMN, default-mode network (Yeo et al., 2011).

Supplementary Table S4. Rank position of ADHD symptoms-related brain regions, relative to the whole-brain distribution of *participation coefficient* p_i and *within-module degree* z_i

**Brain Region**	**BA**	**Hem**	**rank position p_i**	**rank position z_i**
positive association with p_i				
posterior insula*	13	L	68.66	79.43
posterior insula, putamen*	13	R	70.97	79.92
anterior cingulate cortex	24	L	61.91	50.86
superior medial frontal gyrus*	6	L	43.39	42.40
inferior parietal lobe*	40	L	50.75	74.80
negative association with p_i				
anterior cingulate cortex	32, 9	L	43.18	**91.12**
middle frontal gyrus	6	R	31.69	**83.95**
supplementary motor area	8, 6	L	66.32	24.55
posterior fusiform gyrus	20, 36	L	43.03	**4.17**
intraparietal sulcus*	40	R	47.94	78.98
posterior cingulate cortex		R	64.93	**1.31**
middle temporal gyrus	37, 19	R	44.64	52.68
inferior parietal lobe	40	R	35.64	40.59
positive association with z_i				
supplementary motor area	8, 6	R/L	33.31	**12.45**
temporal cortex, amygdala, hippocampus, fusiform gyrus	38, 20, 28	R	52.29	**12.64**
temporal cortex, amygdala, hippocampus, fusiform gyrus	20, 38	L	49.09	**12.38**
precentral gyrus, postcentral gyrus, inferior parietal lobe*	3, 40	R	40.72	**83.64**
precentral gyrus, postcentral gyrus, inferior parietal lobe	3, 40	L	37.86	**82.82**
paracentral lobule	6, 4	L	38.69	23.32
negative association with z_i				
medial frontal gyrus	11, 32, 25	R	50.21	41.27

anterior cingulate cortex	24	R	60.92	57.89
insula, putamen, superior temporal gyrus, inferior frontal gyrus, inferior parietal lobule*	13, 22, 40	L	57.35	71.16
insula, putamen, superior temporal gyrus, inferior frontal gyrus, inferior parietal lobule*	13, 47, 22	R	56.58	67.01
superior medial frontal gyrus*	6, 32, 24	L	52.13	77.52
midde temporal gyrus	21, 20	R	30.66	78.60
thalamus		R	57.32	25.14
thalamus		L	36.58	27.35
posterior fusiform gyrus	37, 19	L	59.31	**17.80**
posterior fusiform gyrus		R	35.77	26.18
posterior cingulate cortex	30	L	57.75	66.01
precuneus	19, 7, 31	R/L	53.49	**81.13**
inferior occipital gyrus	18	R	39.84	36.88

BA, approximate Brodmann's area; Hem, hemisphere; L, left; R, right; regions with significant effects in both measures (participation coefficient and within-module degree) are marked with an asterisk and separately listed in Table 5; rank positions < 20% or > 80% are depicted in bold letters.

Supplementary Table S5. ADHD symptoms and global modularity measures controlled for number of low-motion frames (rather than mean framewise displacement; see Post-Hoc Analyses in the Results Section of the Main Text)

	$r_{part.}$	$p_{part.}$	**BF_{01}-Reg.**
Whole-brain modularity measures			
global modularity	.11	.059	0.63
number of modules	-.09	.148	1.35
average module size	.08	.204	1.89
variability in module size	-.03	.626	3.13
Whole-brain proportions of node types			
ultra-peripheral nodes	.01	.824	3.80
peripheral nodes	.06	.359	2.27
non-hub connector nodes	-.07	.220	2.28
non-hub kinless nodes	-.10	.107	1.02
provincial hubs	.09	.149	1.33
connector hubs	-.07	.253	2.57
kinless hubs	-.07	.263	2.61

$r_{part.}$, Pearson's correlation coefficient for the partial correlation controlling for effects of age, sex, handedness, number of low-motion frames (FD < 0.2mm), and FSIQ; $p_{part.}$, *p*-value of significance for the partial-correlation; BF_{01}-Reg., Bayes Factor in favor of the null hypothesis (i.e., absence of correlation). Bayes Factors were calculated for linear regression models predicting ADHD Index values by the respective whole-brain measure of modular network organization or whole-brain proportions of node types, respectively, while effects of age, sex, handedness, number of low-motion frames (FD < 0.2mm), and FSIQ were controlled.

Supplementary Table S6. ADHD symptoms and participation coefficient controlled for age, sex, handedness, FSIQ, and number of low-motion frames (rather than mean framewise displacement, see Post-Hoc Analyses in the Results Section of the Main Text)

**Brain Region**	**BA**	**Hem**	**x**	**y**	**z**	t_{max}	***k***
positive association							
posterior insula, inferior parietal lobe	13, 40	L	-57	-36	21	5.45	840
posterior inula, putamen	13	R	36	-9	3	3.64	384
anterior cingulate cortex	24	L	-6	18	24	3.60	110
superior medial frontal gyrus	6	L	-18	-3	69	3.32	47
inferior parietal lobe	40	R	63	-30	21	3.18	33
negative association							
middle frontal gyrus	6	R	33	-12	45	2.97	31
supplementary motor area	8, 6	L	-6	18	57	3.87	40
anterior fusiform gyrus	28, 38	R	27	6	-39	3.34	79
anterior fusiform gyrus	20, 36	L	-30	3	-39	3.25	63
posterior fusiform gyrus	20, 37	L	-48	-33	-27	4.09	77
posterior fusiform gyrus	20	R	42	-18	-27	3.70	46
intraparietal sulcus	40	R	33	-36	48	3.38	91
posterior cingulate cortex		R	12	-39	9	3.84	27
middle temporal gyrus	37, 19	R	57	-63	0	3.71	105

BA, approximate Brodmann's area; Hem, hemisphere; L, left; R, right; coordinates refer to the Montreal Neurological Institute template brain (MNI); t_{max}, maximum *t* statistic in the cluster; *k*, cluster size in voxels of size 3 x 3 x 3 mm.

Supplementary Table S7. ADHD symptoms and within-module degree controlled for age, sex, handedness, FSIQ, and number of low-motion frames (rather than mean framewise displacement, see Post-Hoc Analyses in the Results Section of the Main Text)

**Brain Region**	**BA**	**Hem**	**x**	**y**	**z**	t_{max}	***k***
positive association							
supplementary motor area	8, 6	R/L	0	30	60	3.20	42
caudate		R	15	24	-6	3.90	39
temporal cortex, amygdala, hippocampus, fusiform gyrus	38, 20, 28	R	33	6	-30	5.76	794
temporal cortex, amygdala, hippocampus, fusiform gyrus	20, 38	L	-27	-15	-33	5.15	872
precentral gyrus, postcentral gyrus, inferior parietal lobe	3, 40	R	39	-33	51	6.72	588
precentral gyrus, postcentral gyrus, inferior parietal lobe	3, 40	L	-45	-33	51	5.37	373
paracentral lobule	6, 4	L/R	0	-33	72	3.88	180
negative association							
medial frontal gyrus	11, 32, 25	R	3	30	-15	4.42	164
insula, putamen, superior temporal gyrus, inferior frontal gyrus, inferior parietal lobule	13, 22, 40	L	-48	9	-3	5.64	883
insula, putamen, superior temporal gyrus, inferior frontal gyrus, inferior parietal lobule	13, 47, 22	R	39	3	15	5.46	620
superior medial frontal gyrus	6, 32, 24	L	-12	-3	63	5.04	507
midde temporal gyrus	21, 20	R	63	-6	-21	4.48	63
midde temporal gyrus	21, 20	L	-66	-30	-6	3.78	104
cuneus/precuneus	19	R	42	-24	24	3.86	168
posterior fusiform gyrus	37, 19	L	-36	-48	-12	4.26	139
posterior cingulate cortex	30	L	-24	-66	21	3.60	27
precuneus	19, 7, 31	R/L	-12	-87	36	3.84	85
inferior occipital gyrus	18	R	27	-84	-12	3.31	30

BA, approximate Brodmann's area; Hem, hemisphere; L, left; R, right. Coordinates refer to the Montreal Neurological Institute template brain (MNI); t_{max}, maximum t statistic in the cluster; k, cluster size in voxels of size 3 x 3 x 3 mm.

Supplementary References

Yeo, B. T. T., Krienen, F. M., Sepulcre, J., Sabuncu, M. R., Lashkari, D., Hollinshead, M., … Buckner, R. L. (2011). The organization of the human cerebral cortex estimated by intrinsic functional connectivity. *Journal of Neurophysiology, 106*, 1125–1165. http://doi.org/10.1152/jn.00338.2011.
